# Non-hepatic Hyperammonemia Encephalopathy: A Late Complication of Roux-en-Y-Gastric Bypass

**DOI:** 10.7759/cureus.96087

**Published:** 2025-11-04

**Authors:** Oleanna Talia, Syeda Daniya Samreen, Akashdeep Grewal, Syeda J Arshi, Camelia Arsene

**Affiliations:** 1 Internal Medicine, Trinity Health Oakland, Pontiac, USA

**Keywords:** acute hyperammonemic encephalopathy, levocarnitine, non-cirrhotic hyperammonemia, ornithine transcarbamlyase deficiency, roux-en-y gastric bypass (rygb)

## Abstract

Roux-en-Y gastric bypass (RYGB) is a commonly performed bariatric surgery for weight loss in the United States. Despite its widespread use, this invasive surgery is associated with several chronic complications. We present a case of a 37-year-old female who developed hyperammonemic encephalopathy (HE), a rare but potentially life-threatening complication following RYGB. This case adds to existing literature by emphasizing the importance of considering HE as a differential diagnosis in patients presenting with altered mental status post-RYGB and highlights the need for early recognition and effective management strategies to optimize patient outcomes.

## Introduction

In 2018, over 200,000 individuals underwent bariatric surgery after multiple unsuccessful attempts at achieving sustainable weight loss, with an average body weight reduction of approximately 28% following successful surgery [1]. One commonly performed procedure, the Roux-en-Y gastric bypass (RYGB), involves creating a small gastric pouch and attaching it directly to the small intestine, thereby bypassing more than 95% of the stomach [1]. Although highly effective, this invasive surgery is associated with an increased risk of long-term complications due to altered nutrient absorption and metabolism [2].

Hyperammonemic encephalopathy (HE) is a rare but potentially fatal complication associated with a mortality rate of up to 50%. It is characterized by non-cirrhotic hyperammonemia occurring in the absence of underlying liver disease [3]. Clinical manifestations of HE can range from confusion, vomiting, and irritability to seizures, coma, or even death, all due to abnormal changes in mental status and neurological dysfunction. This condition is also marked by increased plasma glutamine levels, low blood albumin levels, reactive hypoglycemia following meals, and deficiencies in essential nutrients [4]. Although the relationship between RYGB and HE remains unclear, proposed mechanisms suggest that this surgical procedure alters the gut microbiome and reduces the availability of essential vitamins, substrates, and metabolizers required for proper urea cycle function, thereby impairing ammonia excretion [5]. In contrast to acquired causes, genetic deficiencies in the urea cycle have also been suspected [5]. Given the significant mortality associated with this complication, early recognition, diagnosis, and intervention are crucial for improving patient outcomes.

## Case presentation

A 37-year-old female with a history of RYGB surgery in 2007, multiple abdominoplasties, poor nutritional intake, gastroesophageal reflux disease (GERD), aortic thrombus, and chronic low back pain managed with opioids presented to the hospital with a one-day history of lethargy and altered mental status.. Upon arrival, she became obtunded, demonstrating minimal eye opening, incomprehensible verbal responses, and flexion to pain, corresponding to a Glasgow Coma Scale (GCS) score of 9, necessitating intubation to secure her airway. The patient was admitted to the ICU for further evaluation and management.

**Figure 1:**
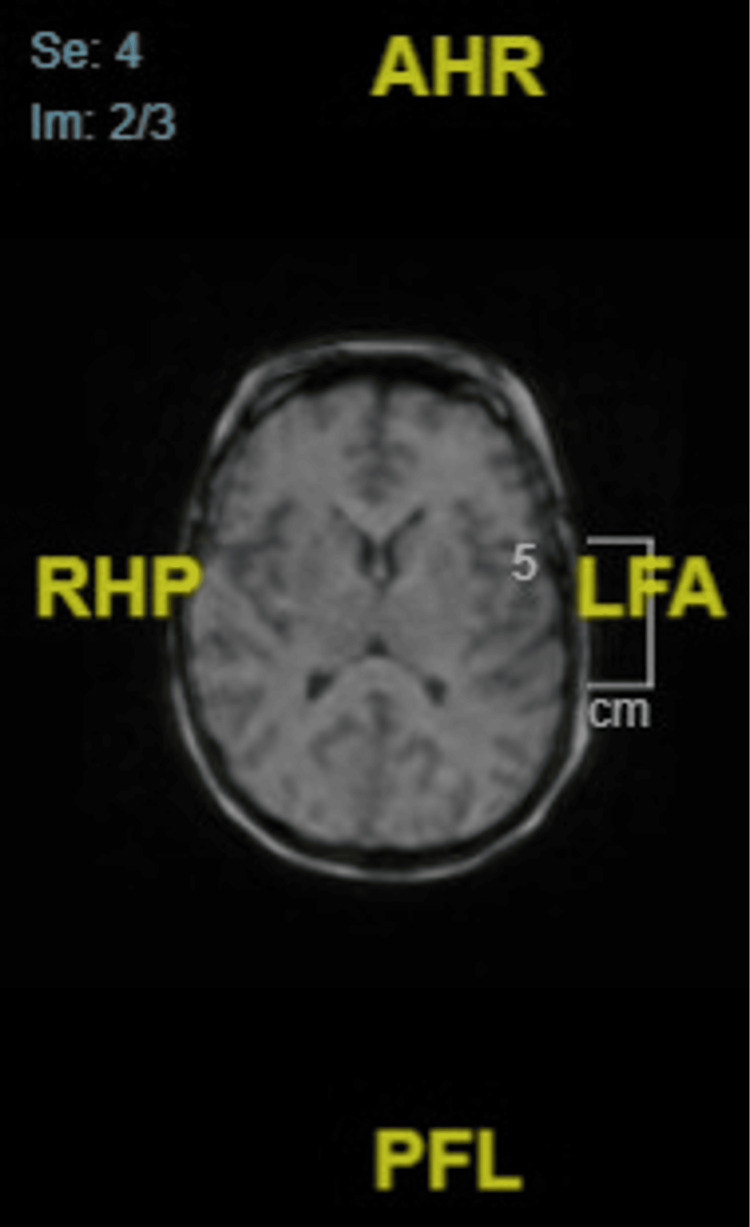
Brain MRI without contrast The patient's brain MRI showed no significant abnormalities or masses MRI: magnetic resonance imaging

**Figure 2:**
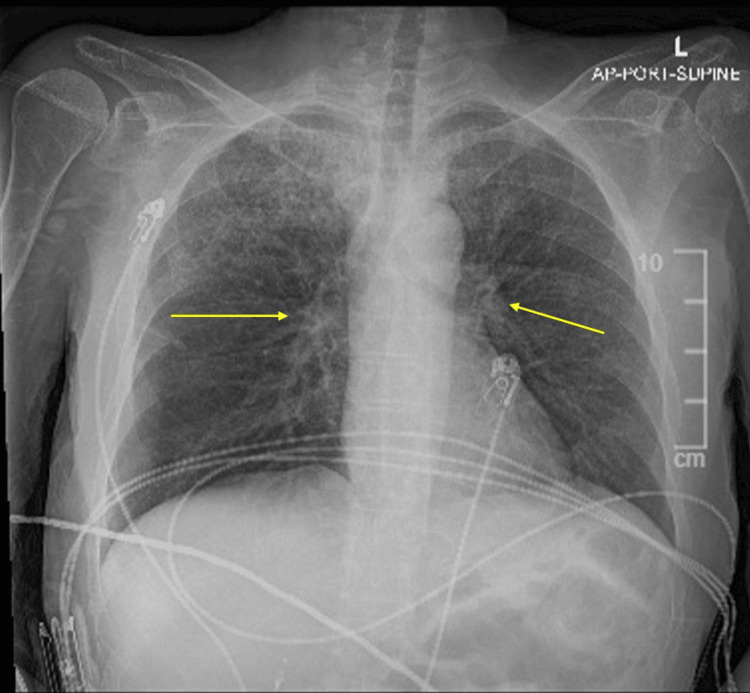
Chest X-ray The patient's chest X-ray showed minimal diffuse interstitial opacities suggestive of atypical pneumonia

Laboratory tests (Table 1) revealed hyperammonemia, elevated thyroid-stimulating hormone (TSH), low thyroxine (T4), hypoglycemia, an elevated white blood cell count, and elevated procalcitonin. An MRI of the head was diffusely unremarkable (Figure 1), while a chest X-ray showed diffuse interstitial opacities suggestive of atypical pneumonia (Figure 2). Additionally, an Electroencephalogram (EEG) demonstrated diffuse theta wave slowing, indicating underlying neurological dysfunction. Vascular Surgery was consulted for a floating thrombus in the descending thoracic aorta, which was managed with IV heparin.

**Table 1:** Laboratory values Patient's labs showed significant hyperammonemia, hypothyroidism, hypoglycemia, leukocytosis, and increased procalcitonin. All other laboratory results were unremarkable TSH: thyroid-stimulating hormone; T4: thyroxine

Lab marker	Patient value	Reference range
Ammonia, µmol/L	82	11-32
TSH, µIU/mL	22.20	0.27-4.2
T4, ng/dL	0.47	0.8-1.8
Glucose, mg/dL	63	70-100
WBC, cells/mL	14.0	4,000-10,500
Procalcitonin, ng/mL	9.87	<0.05

The patient was evaluated for potential causes of acute metabolic encephalopathy, including hyperammonemia, hypoglycemia, myxedema coma, opioid overdose, and sepsis of unknown origin. According to protocol, she was given IV naloxone (2 mg) without improvement. She was then started on IV vancomycin (750 mg every 12 hours) for suspected sepsis and levothyroxine (200 mcg) for myxedema coma. She was subsequently found to be positive for influenza A on a nasopharyngeal respiratory panel, with superimposed bacterial pneumonia. Vancomycin was discontinued, and treatment was adjusted to include methylprednisone, Tamiflu, and piperacillin/tazobactam.

The patient was gradually weaned off sedation, but her mental status did not improve, showing minimal response to noxious stimuli. Repeat ammonia levels consistently increased in the ICU. She was started on lactulose and rifaximin, and Neurology was consulted to further investigate the etiology of hyperammonemia. A plasma amino acid analysis was conducted to explore potential metabolic disorders, including ornithine transcarbamylase (OTC) deficiency. The analysis revealed elevated levels of multiple amino acids, including glutamine, alanine, proline, and glycine.

**Figure 3:**
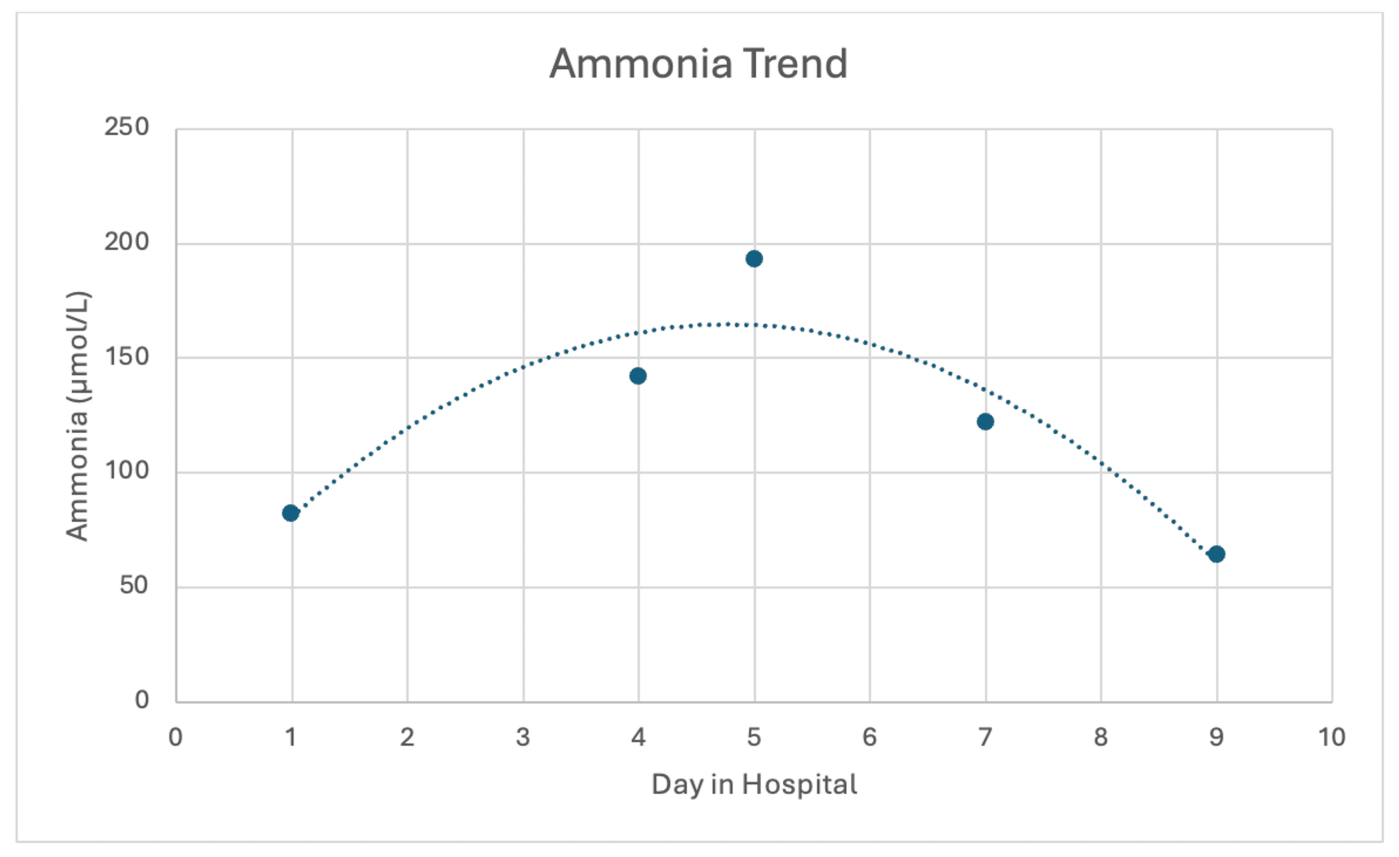
Ammonia level trend Ammonia levels were 82 µmol/L on day 1, rising to a peak of 193 µmol/L on day 5. By day 7, levels began to decline, reaching 64 µmol/L by day 9

Despite appropriate hyperammonemia treatment, the patient’s ammonia levels remained elevated. Based on the amino acid analysis, she was then started on IV levocarnitine (900 mg every six hours) to facilitate alternative nitrogen disposal by forming acylcarnitine conjugates, thereby bypassing a possible acquired urea cycle defect related to her history of RYGB. Following this intervention, ammonia levels continued to decrease, eventually returning to normal (Figure 3). Lactulose and rifaximin were discontinued. As ammonia levels gradually decreased, the patient slowly regained consciousness and began following simple commands. With her improved mental state, she was successfully extubated.

The patient was identified to have multiple micronutrient deficiencies and chronic malnutrition due to RYGB, prompting a consultation with Gastroenterology. They recommended outpatient follow-up with bariatric surgery and resuming lactulose therapy to maintain two to three bowel movements per day. Vascular Surgery advised a repeat CT angiography (CTA), which revealed a smaller thrombus, increasing the risk of plaque rupture. Lifelong anticoagulation with aspirin, apixaban, and clopidogrel was deemed necessary. The patient’s hyperammonemia, encephalopathy, and pneumonia resolved, leading to her discharge from the hospital.

## Discussion

This report highlights a rare presentation of HE in a patient with a history of RYGB surgery. With few cases documented in the literature, the pathophysiological link between hyperammonemia and bariatric surgery remains largely speculative [6]. Proposed mechanisms include both genetic factors (such as urea cycle disorders) and nongenetic factors (such as anatomical and microbiome alterations) [6]. OTC deficiency, one of the most common urea cycle disorders, is an X-linked condition affecting approximately one in 80,000 live births, with 85% of individuals remaining asymptomatic due to X chromosome inactivation [7]. Symptoms can range from mild nausea to severe neurological manifestations following protein intake [7]. Cases of OTC deficiency have been reported after RYGB surgery, attributed to dietary changes and total parenteral nutrition (TPN) aimed at addressing chronic malnutrition, which led to increased protein intake [7]. Similar cases have shown that nutritional stress and prolonged catabolic states are significant contributors to symptom onset in individuals with underlying urea cycle disorders [8].

In patients with OTC deficiency, increased protein levels result in ammonia production, which cannot be efficiently excreted through the urea cycle, leading to hyperammonemia and subsequent encephalopathy [9]. In many instances, symptoms emerged after a triggering event, such as bariatric surgery itself, likely due to the combined effects of inflammatory stress and nutritional deficiencies [10]. The inability to process proteins through the urea cycle causes an accumulation of amino acids in the blood, contributing to further nutritional deficiencies [11]. As in the case presented here, management of the genetic deficiency includes a low-protein diet, TPN, and levocarnitine supplementation [7].

Hyperammonemia can also result from a high-protein diet or prolonged starvation, both of which increase protein metabolism and amino acid deamination, producing ammonia as a byproduct [11]. The accumulation of ammonia impairs the urea cycle’s ability to convert it into urea for excretion, leading to further buildup of ammonia in the bloodstream [11]. The central nervous system, particularly the brain, is highly sensitive to elevated ammonia levels, causing early neurological symptoms such as asterixis, lethargy, confusion, and altered mental status [11]. More severely, hyperammonemia can result in late neurological signs of encephalopathy [11]. Unlike typical management of acquired hyperammonemia, which often relies solely on supportive treatment with lactulose [11], this case highlights that targeted therapy with levocarnitine may be beneficial when an underlying metabolic disturbance is suspected.

## Conclusions

This report underscores the importance of recognizing HE as a potential complication following RYGB surgery, particularly in those with suspected OTC deficiency. Given the poor prognosis, with a mortality rate of 50%, enhancing clinical awareness is essential to improve recognition, understanding, and management of these rare but serious complications. Although it is rare, prevention strategies should emphasize metabolic evaluation, patient education, nutritional counseling, and interdisciplinary follow-up. Understanding the multifactorial triggers following RYGB, ranging from underlying metabolic deficiencies to nutritional and inflammatory stressors, is essential for timely diagnosis and effective management. Early detection and intervention, including dietary modifications, electrolyte correction, and levocarnitine supplementation, are crucial in preventing severe neurological outcomes. Ultimately, multidisciplinary collaboration and increased awareness can significantly improve patient outcomes, prevent life-threatening sequelae, and enhance quality of life in this vulnerable population.
